# Medical Alliance Transactions. Wood County Medical Society. S. S. and G. Association. N. W. Texas Medical Society, Etc.

**Published:** 1891-10

**Authors:** 


For Daniel's Texas Medical Journal.

### Society Notes.

Medical Alliance Transactions.—The Transactions of the first annual meeting of the United States Medical Practitioners' Protective Alliance have been published, and are now ready for distribution to the profession. The volume contains the addresses in full delivered at the Baltimore meeting, together with constitution and by-laws, and other information. A copy will be mailed to any physician interested who will send stamp to the Secretary, Dr. J. F. Davison, Glendola, New Jersey.

---

The Physicians of Wood county have organized a medical society. Meetings are held at Quitman, second Monday in each month. So we aie informed by our subscriber, Dr. Henrie Leath.

---

A Session of the Southern Surgical and Gynecological Association will be held in Richmond, Va., November 10th, 11th and 12th, 1891. President, Louis S. McMurtry, M. D., Louisville, Kentucky; Secretary, W. E. B. Davis, M. D. Birmingham, Ala.

---

The Northwest Texas Medical Society will meet in Cisco the first Tuesday in December. P. C. COLEMAN, M. D.

---

Old Mississippi Heard From.—Mississippi has been the only State, we believe, not represented in the field of medical journalism. The "long felt want" has been supplied. We are in receipt of Vol. 1, No. 1, of "*The Mississipi Medical Monthly*," a journal devoted to the practice of medicine and surgery, published at Meridian, Miss. At the masthead we see the name of Dr. H. H. Harralson, our old time friend and student, and Dr. N. S. Clark, as editors and proprietors. Harralson was with us through the terrible epidemic of yellow fever at Lake, Miss., in 1878, and has got the *nerve* to make a good physician or good editor. We welcome the *Mississippi Medical Monthly*, and wish it *luck*.

---

Splendid Results. — The Antikamnia Chemical Co., St. Louis, says: "We thank you for your kind attentions, and for the splendid results obtained from our advertisement in your journal." That's about what they all say ; send for rates.

---

Club Rates.—Doctor, get DANIEL'S TEXAS MEDICAL JOURNAL and the *New Orleans Medical and Sutgical Journal* both for $3.50; or DANIEL'S TEXAS MEDICAL JOURNAL and *Love's Medical Mirror* for $3.50, for 1892.

